# Extracellular Vesicles as a Novel Liquid Biopsy-Based Diagnosis for the Central Nervous System, Head and Neck, Lung, and Gastrointestinal Cancers: Current and Future Perspectives

**DOI:** 10.3390/cancers13112792

**Published:** 2021-06-03

**Authors:** Anna Testa, Emilio Venturelli, Maria Felice Brizzi

**Affiliations:** Department of Medical Sciences, University of Turin, 10126 Turin, Italy; anna.testa@unito.it (A.T.); emilio.venturelli@edu.unito.it (E.V.)

**Keywords:** extracellular vesicles, cancer, cancer diagnostic biomarkers, liquid biopsy

## Abstract

**Simple Summary:**

To improve clinical outcomes, early diagnosis is mandatory in cancer patients. Several diagnostic approaches have been proposed, however, the main drawback relies on the invasive procedures required. Extracellular vesicles (EVs) are bilayer lipid membrane structures released by almost all cells and transferred to remote sites via the bloodstream. The observation that their cargo reflects the cell of origin has opened a new frontier for non-invasive biomarker discovery in oncology. Moreover, since EVs can be recovered from different body fluids, their impact as a Correctdiagnostic tool has gained particular interest. Hence, in the last decade, several studies using different biological fluids have been performed, showing the valuable contributions of EVs as tumour biomarkers, and their improved diagnostic power when combined with currently available tumour markers. In this review, the most relevant data on the diagnostic relevance of EVs, alone or in combination with the well-established tumour markers, are discussed.

**Abstract:**

Early diagnosis, along with innovative treatment options, are crucial to increase the overall survival of cancer patients. In the last decade, extracellular vesicles (EVs) have gained great interest in biomarker discovery. EVs are bilayer lipid membrane limited structures, released by almost all cell types, including cancer cells. The EV cargo, which consists of RNAs, proteins, DNA, and lipids, directly mirrors the cells of origin. EVs can be recovered from several body fluids, including blood, cerebral spinal fluid (CSF), saliva, and Broncho-Alveolar Lavage Fluid (BALF), by non-invasive or minimally invasive approaches, and are therefore proposed as feasible cancer diagnostic tools. In this review, methodologies for EV isolation and characterization and their impact as diagnostics for the central nervous system, head and neck, lung, and gastrointestinal cancers are outlined. For each of these tumours, recent data on the potential clinical applications of the EV’s unique cargo, alone or in combination with currently available tumour biomarkers, have been deeply discussed.

## 1. Introduction

**Figure 1:**
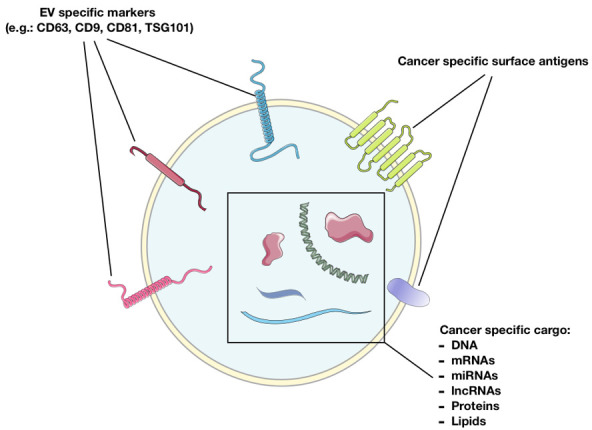
Cancer-derived EV content. Based on the presence of specific EV markers and cancer cell specific markers, EVs may serve as valuable diagnostic tools. CD: Cluster of Differentiation, miRNAs: micro RNAs, lncRNAs: long non-coding RNAs. This figure has been created by Emilio Venturelli using Servier Medical Art templates, which are licensed under a Creative Commons Attribution 3.0 Unported License; https://smart.servier.com (accessed on 14 April 2021) [11].

According to the World Health Organization (WHO), cancer represents the second cause of overall mortality worldwide and accounts for the highest clinical, social, and economic burden across all human diseases [1]. Despite scientific advances in cancer biology, diagnostic techniques, and new therapeutic approaches, the overall survival (OS) of cancer patients remains largely unfulfilling. Some cancers hold low mortality, particularly prostate and thyroid cancer, while others, such as central nervous system (CNS), breast, lung, oesophageal, gastric, and pancreatic cancers, are characterized by worse outcomes, and their metastatic disease is generally fatal [2,3,4,5,6]. More importantly, in several cases, a late diagnosis negatively impacts beneficial treatments, and hence on the OS. Undoubtedly, early cancer diagnosis, through screening programs and better diagnostic tools, is crucial to timely refer patients to effective treatments [7]. Therefore, the identification of new reliable biomarkers is crucial to improve diagnostic accuracy, molecular phenotyping, and even the selection of personalized treatment options. Currently, a great interest has been directed towards non-invasive approaches, such as liquid biopsy. Liquid biopsy refers to the detection of cancer cells and/or cancer cell products/derivatives in body fluids for diagnosis, monitoring, treatment efficacy, and prognosis [8]. Among new cancer derivatives, extracellular vesicles (EVs) are included [9]. EVs are bilayer lipid membrane limited structures, derived from the cell membrane or cytoplasmic materials, released by almost all cell types into the extracellular space. The EV’s cargo is composed of lipids, proteins, amino-acids, DNA, messenger RNAs (mRNAs) and microRNAs (miRNA), and other non-coding RNAs (ncRNAs) (Figure 1). According to the “Minimal information for studies of extracellular vesicles 2018 (MISEV2018) EV subtypes can be classified based on: size (small or medium/large EVs), density (low, medium or high), biochemical composition (CD63+/CD81+- EVs, Annexin A5-stained EVs, etc.), cell culture conditions or cell of origin (podocyte EVs, hypoxic EVs, large oncosomes, apoptotic bodies)” [10].

**Figure 2:**
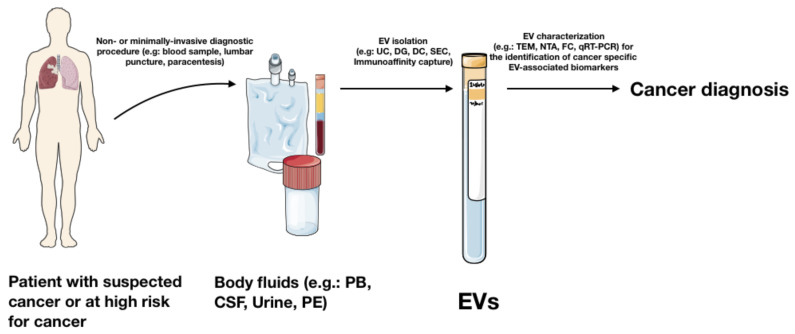
EVs as a diagnostic tool in clinical practices. UC: Ultracentrifugation, DG: Differential Gradient Centrifugation, DC: Differential centrifugation, SEC: Size Exclusion Chromatography, TEM: Transmission Electron Microscopy, NTA: Nanoparticle Tracking Analysis, FC: Flow Cytometry, qRT-PCR: quantitative Real Time Polymerase Chain Reaction, PB: Peripheral Blood, CSF: Cerebrospinal fluid, PE: Pleural effusion. This figure has been created by Emilio Venturelli using Servier Medical Art templates, which are licensed under a Creative Commons Attribution 3.0 Unported License; https://smart.servier.com (accessed on 14 April 2021) [11].

EVs participate in several biological processes both in physiological and pathological human diseases. Specifically in cancer, EVs play an essential role in different oncogenic processes, including cell-to-cell communication, the shaping of the tumour microenvironment (TME), epithelial–mesenchymal transition, and pre-metastatic niche formation [12,13]. The EV cargo frequently differs between tumour- and healthy-derived EVs. EVs can be recovered from several body fluids, and in particular from blood, urine, Broncho-Alveolar Lavage Fluid (BALF), ascites, and cerebrospinal fluid (CSF). Most importantly, these fluids can be easily obtained using non-invasive or minimally invasive procedures, thus representing a relevant diagnostic tool (Figure 2).

Given these peculiarities, EVs hold great potential as cancer biomarkers and diagnostics. In this review, evidence on EVs, as principal and/or ancillary diagnostic biomarkers, in the most clinically relevant neoplastic diseases involving the CNS, head and neck, lung, and the gastrointestinal tract, are discussed.

## 2. Isolation and Purification Methods for EV Clinical Application

EVs can be isolated using different techniques. Ultracentrifugation (UC) or Differential Centrifugation (DC), density gradient ultrafiltration (DG), size exclusion chromatography (SEC), precipitation, immunoaffinity capture, and microfluidic-based techniques are the currently employed in EV isolation methodologies [14]. These methods are summarized in Table 1 and reviewed in [15].

However, no consensus exists on the best, most specific, and appropriate isolation method for clinical application. Therefore, larger hospital-based studies are needed to assess which method is more suitable.

The transfer of EVs in clinical settings has also been proposed. It has been demonstrated that EVs can be obtained from different biological fluids, such as serum, plasma, urine, and BALF. Saenz-Cuesta and colleagues [21] compared ExoQuick isolation, UC, and DC and proposed the DC protocol for the clinical practice. The improvement, refinement, and standardization of similar protocols should match well with the workload of diagnostic laboratories, and therefore, be more feasible for clinical purposes.

After isolation, EV characterization and analysis is required for diagnostic application. This step should confirm the isolation accuracy and the identification of cancer-specific biomarkers with diagnostic potential. TEM is widely used in the EV research field [21,22]. It allows the definition of size, structure, and EV shape. The use of gold nanoparticle conjugation with antibodies is also suitable to detect EV-bond-specific markers, such as Clusters of Differentiations (CD). Similarly to TEM, Atomic Force Microscopy (AMF) is appropriate to assess EV structure and allows the identification of surface markers [23,24]. However, both TEM and AFM cannot provide information on either EV number or cargo (e.g., mRNA, miRNA, DNA, lncRNA). Moreover, TEM and AMF require dedicated and trained personnel. Diffuse Light Scattering (DLS) and Nanoparticle Tracking Analysis (NTA) are also essential for EV basic research. They allow a fast, easy, and reliable analysis of EV number and size distribution [25]. Flow Cytometry (FC) holds great potential in EV characterization and is generally available in both research and clinical departments. Standard FC can help EV phenotyping based on their surface antigen, however, is unsuitable for their quantification. Conversely, new generation flow cytometers can detect particles as small as 150 nm and can be used for both profiling and quantification of most, but not all, EVs [26,27].

The detection of protein markers associated with EVs can be obtained by the conventional Western Blot (WB) technique. It can be used to rapidly validate the presence of EVs in clinical samples by searching for specific EV markers (such as CD63, CD81, TSG101, and CD9) [28]. It must be noted that to avoid the use of contaminated material (for example, circulating free proteins) the WB must be performed on purified EVs. Again, WB analysis does not allow for their quantification. An enzyme-linked immune-adsorbent protein assay (ELISA) is considered a useful method for EV protein quantification and detection [29]. However, both ELISA and its variants do not allow EV structure and shape characterization as well as their quantification.

All the aforementioned methods are exploited for EV characterization. However, they do not allow EV cargo profiling, which is relevant for both biological and diagnostic purposes. Quantitative real-time polymerase chain reaction (qRT-PCR) is less sensitive given the low content of nucleic acids in EVs. It has been shown that digital PCR can potentially overcome this hurdle and correctly detect even low expressed RNAs and DNA. Moreover, digital PCR offers the opportunity to assess the absolute quantitation of transcripts and to estimate the DNA copy number within EVs. Therefore, digital PCR represents a useful approach to detect and quantify EV-derived DNA, RNAs, and microRNAs, and to analyse the mutational status of specific genes in EVs isolated from body fluids in clinical settings [26,30,31]. Finally, more recently, OMIC and Multi-OMIC techniques have gained popularity in translational research, holding great promises in the setting of precision medicine, primarily in oncology. These include genome sequencing, proteomics, transcriptomics, and lipidomics [26,32,33]. The discussed methods are in Table 2.

Unfortunately, no standardized diagnostic protocols are currently available for the diagnostic implementation of EVs in clinical oncology. Ideally, cancer-type-specific protocols should be developed as suggested by Jakobsen et al. [34] in lung cancer. In this regard, the authors applied an ELISA-based EV-array to detect EV protein content demonstrating the feasibility (rapid, automated, and economic) of such methodology for EV-based diagnosis. Interestingly, an appropriate diagnostic accuracy was obtained using a small sample volume (10 μL). Similarly, Sun and colleagues [35] isolated EVs and applied WB and ELISA for Copine III (CPNE3) detection in Colorectal Cancer (CRC) patients. The authors were able to obtain an adequate EV protein content displaying a high diagnostic specificity (cut-off set at 0.143 pg per 1 µg of EVs) starting from 0.5 mL plasma.

A potential diagnostic algorithm using EVs from Hepatocellular Carcinoma (HCC) cells, was also suggested by Julich-Heartel et al. [36]. They showed that EVs with high diagnostic efficacy could be obtained from 1.5 mL of serum using Fluorescent-Activated Cell Scanning (FACS) protein profiling.

## 3. Tumours of the Central Nervous System (CNS)

The definitive diagnosis of brain cancers still depends on biopsy [37], which is not devoid of procedural risks and usually requires multiple sample collections [38]. Furthermore, tissue analysis takes a representative picture of the disease at the time of tissue collection. Hence, the need for novel minimally invasive diagnostic tools for brain tumours can be considered as still unmet [38]. Indeed, EVs have been considered promising biomarkers in neuro-oncology [39,40,41]. The most relevant evidence on EVs as a diagnostic instrument in CNS-derived tumours are discussed.

### 3.1. Gliomas

Diffuse gliomas are the most frequent primary brain malignant tumours [42]. Glioma cells can produce different types of EVs, including apoptotic bodies and oncosomes. The latter consists of atypically large vesicles selectively produced by tumour cells [43]. EVs can pass through the blood–brain barrier (BBB), both in physiological and pathological conditions, and can be therefore detected in both the bloodstream and CSF [44]. Both nucleic acids and proteins can be found in serum-derived EVs [45]. It has been shown that serum EVs derived from patients with glioblastoma (GBM) carry a 60-fold greater amount of RNAs compared to free-circulating RNAs recovered from the whole blood, plasma, or serum [46] (Table 3).

Moreover, it has been reported that the phosphorylated and bioactive mutant EGRFvIII variant (EGRFvIII) is enriched in EVs released from GBM [64]. In the pioneer study, Skog et al. [52] demonstrated the presence of the EGRFvIII mRNA in 7 out of 25 patients suffering from GBM. Interestingly, the surgical removal of the primary lesion translated into the loss of the EGRFvIII variant in the patients’ plasma. This was the first description of the EGFRvIII transcript in EVs derived from viable cancer cells. In a different study, Manda et al. [53] focused on the expression of both EGFR and EGFRvIII in serum-derived EVs and tumour tissues in 96 patients with high-grade gliomas. This study identified EGFRvIII in 39.5% of the tumour samples and 44.7% of the serum EV samples, whereas only 28.1% of the tumor biopsies co-expressed EGFR and EGFRvIII. They also demonstrated that EGFRvIII EV content correlates with a poor OS—21.1 months—versus 28.6 months when compared to patients without EGFRvIII EV enrichment. However, since EGFRvIII is only detectable in ~25% of glioma patients its value as a biomarker may be limited [65]. Recently, Chandran et al. [54] demonstrated that circulating EVs enriched in syndecan-1 (SDC1) discriminate low-grade gliomas from GBM with a sensitivity of 71% and a specificity of 80%. Furthermore, the observation that circulating EVs enriched in SDC1 correlated with the expression of SDC1 in matched tumors, and decreased after surgical removal, provided a strong support that SDC1-EV content comes from GBM tumours (Table 3).

Yang et al. [55] isolated circulating EVs from xenograft mice bearing GBM-patient derived xenograft (PDX), and EVs from controls. Data from the mRNA profiling identified three upregulated genes in EVs from xenograft mice corresponding to p65, DNM3, and CD117, and three downregulated genes matching with PTEN, p53, and APC. Lan et al. [47] identified a significant difference in EV-miR-301a content while comparing 60 glioma patients and 43 patients without glioma. The miR-301a expression was significantly high in both high- and low-grade glioma patients, underwent reduction after surgical resection, and further increased upon recurrence (Table 3). Ebrahimkhani et al. [48] have used a panel of seven EV-miRNAs (miR-182-5p, miR-328-3p, miR-339-5p, miR-340-5p, miR-485-3p, miR-486-5p, and miR-543) to discriminate GBM patients from healthy controls with an accuracy of 91.7%. Manterola et al. [49] analyzed the EV-miRNA signature using sera from 75 patients with GBM (381 miRNAs were screened). The authors concluded that miR-320 and miR-574-3p, as well as RNU6-1, were upregulated and able to distinguish GBM patients from healthy controls. The Area Under the Curve (AUC) corresponded to 0.926 for the three ncRNAs, 0.852 for RNU6-1, 0.720 for miR-320, and 0.738 for miR-574-3p. This suggested that either RNU6-1 alone or combined with miR-320 and miR-574-3p displays a diagnostic significance.

In addition to the RNA or DNA, EVs also harbour cellular proteins on their surface, reflecting their cell of origin [66]. Shao et al. [59] identified a panel of four proteins on the EV surface (EGFR, EGFRvIII, PDPN, and IDH1 R132H), which differentiate GBM patients from controls, by using an antibody capture-based technique and a miniaturized nuclear magnetic resonance system (µNMR). This technique requires small sample volumes (1 μL) without the need for extensive purification or a time-consuming approach. The detection sensitivity far surpassed other analytical methods, with a threshold of ~10^4^ EVs (corresponding to ~0.02 ng of protein). The accuracy of each marker, obtained from the Receiver Operating Characteristic (ROC) curves, was <76% and became >90% when the four markers were combined. Huang et al., [60] besides demonstrating that the Polymerase I and the transcript release factor (PTRF) are downstream to EGFRvIII, analyzed tumour samples and matched blood samples from 18 WHO grade II and 18 WHO grade IV patients. They have shown a higher PTRF/CD63 ratio at the protein level in WHO grade IV samples both in tumour tissues and in circulating EVs. Furthermore, they demonstrated that PTRF/CD63 ratio was significantly decreased one week after surgery in circulating EVs and that the PTRF/CD63 ratio can predict EV secretion. Based on these observations they proposed that the PTRF/CD63 ratio as a glioma diagnostic and prognostic biomarker [62].

Osti et al. [61] identified the so-called “GBM EV protein signature” by comparing different GBM EV samples. Indeed, they demonstrated the presence of 11 differentially expressed EV-associated proteins (vWF, APCS, C4B, AMBP, APOD, AZGP1, C4BPB, Serpin3, FTL, C3, and APOE) [67,68,69,70,71,72,73,74] (Table 3). Furthermore, since the loss of their expression was reported after surgery, the authors proposed these proteins to distinguish GBM patients from healthy controls. All of the above studies proposed EVs as diagnostic biomarkers based on their specific cargo. Conversely, the EV number, which reflects tumour occurrence and the response to treatment, was suggested for the follow-up of GBM by Osti et al. [61]. Similarly, Andre-Gregoire et al. [75] have reported a higher EV number in the plasma of GBM patients than in the controls’. Overall, these data indicate that EVs carrying predictive GBM markers would be of particular interest as non-invasive diagnostic and prognostic tools, better in combination [76].

The number of studies focusing on EV-derived lipids as cancer diagnostic biomarkers is limited. Haraszy et al. [77] performed a lipidomic analysis of EVs derived from two different cell lines, U87 glioblastoma cells and Huh7 hepatocellular carcinoma cells, and on human bone-marrow-derived mesenchymal stem cells (MSCs). The Huh7 and MSC-EV lipidomes were similar to each other and they significantly differed from the U87-derived EVs. In particular, the Huh7 and MSC-EVs were specifically enriched in cardiolipins, while the U87 in sphingomyelins. Further studies are required to assess the role of EV lipid content in glioma diagnosis.

CSF is also considered a viable biofluid to isolate and characterize EVs. Indeed, Chen et al. [56] have shown that CSF, unlike plasma, contains EVs enriched in the mutant Isocitrate Dehydrogenase (IDH1) mRNA, which displays a 63% sensitivity and a 100% specificity in patients suffering from gliomas. Figueroa et al. [78] demonstrated that EVs carrying the oncogenic EGFRvIII mRNA isolated from the CSF are more sensitive and specific (60% sensitivity and more than 98% specificity) than the gold standard (qPCR) on brain tumor tissues. Akers et al. [79] have also shown that miRNA profiling of EVs from CSF can distinguish tumours and non-tumoural diseases. In particular, the authors demonstrated that the level of miR-21 in EVs isolated from the CSF of GBM patients was 10-fold higher than in controls. Furthermore, the up-regulation of miR-21, miR-218, miR-193b, miR-331, and miR-374a and the downregulation of miR-548c, miR-520f, miR-27b, and miR-130b have been proposed as a “CSF miRNA signature” in GBM. As a matter of fact, when prospectively applied to cisterna and lumbar CSF, the sensitivity and specificity of the “CSF miRNA signature” corresponded to 67% and 80%, and 28% and 95%, respectively [50]. However, the risks associated with CSF lumbar sampling may prevent its routine application in clinical practice [80].

### 3.2. Brain Metastases, Nonfunctional Pituitary Adenomas, Pediatric Brain Tumours

The poor prognosis associated with metastatic tumours reflects the great need for specific early biomarkers in this setting [81]. Santangelo et al. [51] focused on the miRNA signature of serum-derived EVs in glioma patients, to intending to differentiate tumour grading and gliomas from brain metastases. The upregulation of miR-21, miR-222, and miR-124-3p was reported in gliomas. Interestingly, miR-21, known to play a key role in the pathogenesis of GBM [82], also discriminates healthy controls from glioma patients, but not high-grade gliomas from brain metastases. Nevertheless, when combined in a panel, including miR-222 and miR-124-3p it was able to distinguish high-grade gliomas from brain metastases. This panel has been therefore proposed as an alternative diagnostic tool in patients with no diagnostic biopsy or with tumours located in critical brain areas [51].

Wang et al. [63] focused on nonfunctional pituitary adenomas (NFPAs) and demonstrated a lower expressions of folate receptor 1 (FOLR1) and epithelial cell adhesion molecules (EpCAM) in serum EVs derived from 10 patients suffering from invasive NFPAs compared to 10 healthy controls. Both FOLR1 and EpCAM displayed outstanding sensitivity and specificity in discriminating invasive and noninvasive NFPAs: AUC 0.940, 95% confidence interval (CI) 0.8331 for FLOR1, and AUC 0.880, 95% CI 0.7278 for EpCAM. The ROC analysis showed that the EV protein content failed to discriminate invasive NFPAs from noninvasive ones [63]. In addition, increased vimentin and N-cadherin mRNA contents were found in the serum EVs from patients with invasive NFPAs compared to noninvasive ones. The presence of epithelial–mesenchymal transition (EMT) markers in the serum EVs have suggested the potential application of serum EVs to evaluate the EMT reprogramming of pituitary adenoma and therefore for the diagnosis of invasive NFPAs. The authors compared the transforming gene 1 (PTTG1) mRNA expression in 11 noninvasive and 11 invasive NFPA patients and found a significantly high PTTG1 mRNA content in the invasive group. These results point to the relevance of using EVs not only for a noninvasive diagnostic approach, but also to predict prognosis, and surgical risk in NFPA patients [63].

The relevance of EVs as biomarkers for a minimally invasive diagnosis is even more evident for pediatric brain tumours. Likewise for the mutant form of BRAF (V600E) detected in EVs collected from the plasma of melanoma patients [83], D’asti et al. [57] recently reported that EVs released from cultured juvenile pilocytic astrocytoma (JPA) cells are enriched in KIAA1549/BRAF fusion transcript. Jackson et al. [58] have also isolated EVs from metastatic and non-metastatic medulloblastoma cell lines. By analyzing their cargo, they demonstrated that the metastatic cell lines released an increased number of EVs compared with non-metastatic ones and that these EVs are enriched in mRNAs of metastatic-associated genes such as c-Met, ABCB1, MMP2, BSG, and ITG-A9. They proposed EVs as a potential diagnostic biomarker in patients suffering from medulloblastoma (Table 3).

## 4. Head and Neck Cancers

### 4.1. Oral Cancer

Saliva is considered an easily available and noninvasive source for early biomarker detection in high-risk oral cancer (OC) patients [84]. Glandular saliva better reflects the physiology of the major salivary glands and is much “cleaner” than the whole saliva, consisting of a complex mix of fluid from major and minor salivary glands and gingival crevicular fluid. On the contrary, the whole saliva is a more appropriate source for biomarker detection of different OCs, as malignant EVs can be recovered from the whole saliva simply by bathing the oral cavity [85]. Zlotogorski-Hurvitz et al. [84] have demonstrated that the oral fluid from OC patients contains EVs that differ morphologically and molecularly from those obtained by oral fluids of OC-free individuals. In particular, the authors concluded that the significantly decreased expression of CD9 and CD81 on EVs could serve as an OC marker, even at the early stage of the disease. Gai et al. [86] analyzed 21 patients with oral squamous cell carcinoma (OSCC) and 11 controls, by isolating EVs from saliva. They identified two miRNAs (miR-412-3p and miR-512-3p) overexpressed in OSCC patients. These mRNAs showed a discrimination power with high sensitivity and specificity and the AUC values corresponding to 0.871 and 0.847 respectively. The *p* values were lower than 0.02. Furthermore, two miRNAs (miR-302b-3p and miR-517b-3p) were found selectively enriched in EVs from OSCC patients. These four miRNAs have therefore been proposed as OSCC biomarkers [86]. Interestingly, a previous study identified the upregulation of circulating miR-494 and miR-3651 and the downregulation of miR-186 as relevant biomarkers in 57 OSCC patients. Moreover, since these mRNAs significantly correlated with OSCC, the authors also proposed the dysregulated miRNAs as specific biomarkers [87].

In a different study, salivary EVs enriched in miR-24-3p were selected among three upregulated mRNAs in OSCC. These miRNAs have been proposed as promising diagnostic biomarkers. The analysis of saliva samples from 45 OSCC patients and 10 healthy controls was able to define the discrimination power of EV-miR-24-3p content for OSCC with a sensitivity of 64.4% and a specificity of 80% and AUC corresponding to 0.738. Furthermore, the authors demonstrated that miR-24-3p is involved in the control of OSCC cell proliferation. They concluded that EV-miR-24-3p content may serve as both a clinical noninvasive salivary diagnostic biomarker and a novel OSCC therapeutic target [88]. Notably, previous studies have shown that miR-24-3p is highly expressed in head and neck squamous cell carcinoma and exerts oncogenic functions [89,90,91]. However, the specificity of miR-24-3p for the diagnosis of OSCC is controversial since the abnormal expression of miR-24-3p was also found in several cancers [88]. Numerous studies have also identified salivary EV-mRNA content as potential biomarkers for OSCC. Momen-Heravi et al. [92] have reported that miRNA-27b is significantly upregulated in EVs recovered from the saliva of OSCC patients. The authors have shown its high sensitivity and specificity in detecting OSCC compared to other miRNAs, and its efficacy in distinguishing OSCC patients in remission and individuals with oral lichen planus. A different study found that the salivary miR-31 was significantly high in all OSCC stages regardless of the tumour size. Moreover, the level of miR-31 was higher in saliva than in plasma, suggesting a local production of miR-31. This notion was strengthened by the observation that surgery substantially reduced its salivary content [93]. Finally, Zahran et al. [94] reported a significant increase in salivary EV-miR-21 and miR-184 and a significant decrease of EV-miR-145 in OSCC patients when compared to healthy subjects and patients with recurrent aphthous stomatitis. Moreover, they demonstrated that EV-miR-184 content discriminates OSCC from different oral malignant disorders (Table 4).

### 4.2. Nasopharyngeal and Oropharyngeal Squamous Cell Carcinoma

Liu et al. [97] have demonstrated the overexpression of cyclophilin A (CYPA) in sera, tissues, and circulating EVs of patients suffering from nasopharyngeal cancers (NPCs). In particular, the ROC curves exploited to analyse the diagnostic values of the whole sera and the EV-CYPA content demonstrated that the AUCs correspond to 0.631 (CYPA detected from the whole sera; *p* = 0.042) and 0.844 (EV-CYPA; *p* < 0.0001), indicating that EV-CYPA enrichment has a higher diagnostic significance than the serum CYPA content. Moreover, the relatively low positive rate of EBV-VCA-IgA in NPC patients provided the rationale to investigate the combination of these two markers for the diagnosis of NPCs. They demonstrated that a combination of EV-CYPA content and EBV-VCA-IgA increases the diagnostic accuracy, particularly in patients negative for EBV-VCA-IgA. More recently, Zou et al. [96] identified a useful diagnostic miRNA signature in the serum of NPC patients. They demonstrated that let-7b-5p, miR-140-3p, miR-192-5p, miR-223-3p, and miR-24-3p, combined into a panel, show a high probability to predict NPC. The AUCs corresponding to 0.910 (95% CI: 0.841–0.979) for the training stage, 0.916 (95% CI: 0.886–0.947) for the testing stage, 0.968 (95% CI: 0.936–1.000) for the external validation stage, and 0.912 (95% CI: 0.886–0.937) for the combined three stages were reported (Table 4). Nguyen et al. [98] originally described the presence of circulating HPV in EV-DNA/RNA in patients with HPV-oropharyngeal squamous cell carcinoma (OPCSCC). The authors detected baseline circulating HPV-DNA in cf-DNA from 21 out of 23 HPV-OPCSCC cases (91% sensitivity), while the HPV-DNA in circulating EVs was only detectable in 8 out of 19 patients (42% sensitivity). Similarly, the detection of circulating tumour-derived HPV-RNA was significantly higher in cf-RNA compared to EV-RNA (*p* = 0.0019), confirming that cf-DNA is superior to EV-DNA for HPV-OPCSCC diagnosis (Table 4).

### 4.3. Laryngeal Squamous Cell Carcinoma

Wang et al. [95] compared the EV-miR-21 and HOTAIR content in the serum of patients suffering from laryngeal squamous cell carcinoma (LSCC) and polyps of the vocal cords. They demonstrated that EV-miR-21 and HOTAIR content was significantly higher in patients with LSCC than in patients with vocal cord polyps. Combining EV-miR-21 and HOTAIR, the area under the ROC curve corresponded to 87.6%, which was significantly higher than 80.1% (*p* = 0.0359) and 72.7% for HOTAIR (*p* = 0.0012), and miR-21 alone. Overall, the combo was able to differentiate malignant neoplasms from benign laryngeal disease with 94.2 and 73.5% of sensitivity and specificity, respectively. Furthermore, a significant difference was found among advanced T classifications (T3/T4), clinical stages (III/IV), and the early stages [95]. More recently, Shimada et al. [99] focused on the identification of markers allowing a differential diagnosis among lung squamous cell carcinoma (LSQCC), solitary metastatic lung tumour (MSQCC) and head and neck squamous cell carcinoma (HNSQCC). The validation dataset with formalin-fixed paraffin-embedded (FFPE) from MSQCC and LSQCC demonstrated that miR-10a, miR-28, miR-141, and miR-3120 enriched in circulating EVs were significantly higher in the LSQCCs than the MSQCCs and HNSQCC, while the expression of the same miRNAs in circulating EVs from the LSQCC patients was significantly higher than those from the MSQCC patients. However, the levels of the four EV-miRNAs from four LSQCC cell lines and one HNSQCC cell line showed no significant differences in the expression between the two subtypes, except for miR-3120 (Table 4).

## 5. Lung Cancers

Lung cancer is one of the most common malignancies worldwide. In 2021, in the U.S., nearly 235,760 new lung and bronchus cancer cases and 131,889 deaths have been estimated. Considering its high incidence and its relatively poor prognosis, it accounts for 22% of cancer-related deaths in both males and females [100]. According to the WHO classification, lung carcinoma includes adenocarcinoma, squamous cell carcinoma (SCC), and neuroendocrine tumours (NETs) [101]. Among thoracic NETs, low and intermediate grade tumours (typical and atypical carcinoids) and high grade, highly aggressive cancers such as Small Cell Lung Cancer (SCLC) and Large Cell Neuroendocrine Carcinoma (LCNEC) have been included. Based on differences in the approved treatment options, Non-Small Cell Lung Cancer (NSCLC), including adenocarcinoma and SCC, and Small Cell Lung Cancer (SCLC) have been clinically distinguished. Moreover, as for several malignancies, lung cancer is considered a heterogeneous disease, displaying different molecular profiles. The mutation of the K-RAS, EGFR, ALK/EML-1, c-MET, TP-53, and Rb genes are the recurrent genetic alterations detected in lung carcinoma and are therefore widely considered as targets for molecular-based therapies. However, in clinical practice up to two-thirds of lung cancers are diagnosed at stage III (locally advanced) or VI (metastatic cancers) which constricts the therapeutic success [3]. This implies that, again, early diagnosis represents a challenge for up-to-date oncology. EV-related mRNAs and proteins can be recovered from serum, plasma, BALF, or pleural effusion (PE). These biological fluids can be easily obtained both in inpatient or outpatient settings and their use was found to be feasible for EV isolation in NSCLC patients [102]. Aushev and colleagues [103] identified six miRNAs that can be considered potential biomarkers by comparing serum free and EV-associated miRNAs in patients with lung SCC before and after surgical resection. Originally, the drop in serum free miRNA levels after surgery allowed the identification of miR-205, miR-19a, miR-19b, miR-451, miR-30b, and miR-20a as specific for SCC. However, the strongest evidence came from miR-205 since it dropped in SCC patients, unlike in controls. In the same study, the authors compared the miRNA expression profile in the plasma of patients before and after EV depletion. Interestingly, plasma EVs showed a significant enrichment in miRNA content. For lung adenocarcinoma, the comparison between arterial and peripheral plasma miRNAs led to the identification of six miRNA (miR-19-3p, miR-21-5p, miR-221-3p, miR-409-3p, and miR-425-5p) significantly overexpressed in patients compared with controls [104]. According to the authors, the six-miRNA panel has a specificity and sensitivity of 73% and 80%, respectively. However, when the EV-associated miRNAs were considered, only miR-19-3p, miR-21-5p, and miR-221-3p were found to be significantly upregulated in lung cancer patients. In particular, EV miR-21 content has been proposed as a potential biomarker for other cancers such as oesophageal and laryngeal squamous cell carcinoma. Additional serum derived-EV-based strategies have therefore been proposed. Cazzoli et al. [105] have proposed a preliminary screening and a diagnostic test for lung adenocarcinoma. They performed a wide range analyses on 278 miRNAs in patients with lung adenocarcinoma, granuloma, and healthy controls (former smokers). Fourteen miRNAs were selected for further investigation: miR-502-5p, miR-376a-5p, miR-1974, miR-378a, miR-379, miR-151a-5p, miR-139-5p, miR-200b-5p, miR-190b, miR-30a-3p, miR-629, miR-17, miR-100, and miR-154-3p. In lung adenocarcinoma patients, all miRNAs were upregulated, while in lung granulomas a slight downregulation of miR-139-5p, miR-30a-3p, and miR-378a was detected. Based on their different level of expression, a specificity and a sensitivity of 97.5% and 72% for the screening test and 96% and 60% for the diagnostic test were reported, respectively. The upregulation of the anti-apoptotic miRNA, miR-505-5p, was found in the serum of patients with lung adenocarcinoma. The downregulated miR-382-3p in lung adenocarcinoma patients compared with healthy controls displayed an 85.7% sensitivity and a 95.8% specificity [106]. Recently, Zhong et al. [107] have identified miR-520c-3p and miR-1274b as potential early diagnostic NSCLC biomarkers. These two miRNAs were found to be significantly increased in stage I (according to the TNM staging system) NSCLC patients compared to the control group. Interestingly, the bulk of miR-520c-3p and miR-1274b was packaged in circulating EVs. As shown by Jakobsen et al. [34], EV-associated proteins are also exploited, alone or in combination, with EV associated miRNAs as lung cancer biomarkers. A 30-marker platform, including TAG72, MUC, CD 142, N-cadherin, EGFRvIII, and CD163 combined with the EV markers, CD9, CD63, CD8, TSG101, Hsp90, and HeCam demonstrated a sensitivity of 75% and a specificity of 76% (Table 5).

Only a few studies tried to identify EV-associated lipids as cancer biomarkers. Among them, Fan et al. [108] performed the lipid profiles of EVs obtained from normal and NSCLC subjects and applied two multivariate statistical methods, the Random Forest (RF) and the Least Absolute Shrinkage and Selection Operator (LASSO), to select 23 lipids. The authors were able to discriminate between early- and late-stage cancers and controls (AUC corresponding to 0.85 and 0.88 for RF and 0.79 and 0.77 for LASSO, respectively).

PE is a relatively common clinical presentation of lung cancer [109]. In this clinical setting, pleural fluid can be easily obtained and analysed. EVs are present in the PE and may serve for the differential diagnosis of lung cancers. Lin et al. [110] have compared EV-associated miRNAs in the PE of patients suffering from pneumonia, pulmonary tuberculosis, and lung cancers. The EV-associated miRNAs showed different profiling in the cancer group when compared with the others. Different studies investigated the potential of EV-associated miRNAs to discriminate malignant from non-neoplastic PE. It has been reported that miR-1-3p and miR-144-5p [111] are downregulated, while miR-150-5p [112], miR-182, and miR-210 [113] are upregulated in malignant PE. Therefore, they proposed these miRNAs as diagnostics (Table 5). The potential application of a screening program for lung cancer using a low-dose CT scan in a high-risk smoker is currently available and has been proposed as a part of the clinical practice [114]. However, the high false positives represent the major pitfall of using this procedure. Therefore, the combination of radiological screening (low dose CT) and EV-based strategies would represent the future challenge to reduce the rate of false positive results and allow for greater accuracy in lung cancer screening.

## 6. Cancers of the Gastrointestinal Tract

### 6.1. Oesophageal Cancer

Among gastrointestinal malignancies, the oesophageal cancer is unique. It embodies two distinct major histopathologic types: squamous cell carcinoma (ESCC) and adenocarcinoma (EAC) [115]. Other malignancies involving the oesophagus, such as primary oesophageal melanoma, leiomyosarcoma, or lymphoma are much rarer than the ESCC and adenocarcinoma. The epidemiology of oesophageal carcinoma widely differs worldwide. Indeed, some geographic areas display a 20 to 30-fold increased incidence [100,116]. Regarding the pathology of the oesophageal carcinoma, EAC represents the most common histological subtype. Endoscopy and biopsy, with or without lesion brushing, are commonly used for the diagnosis, displaying a diagnostic accuracy close to 100% [117]. However, it is generally diagnosed late and has a poor prognosis, with a 5-year OS rate corresponding to 20% [118]. Non-invasive diagnostic and screening protocols, offered to high-risk individuals (e.g., familial history of oesophageal carcinoma, smokers, heavy alcohol drinkers), might therefore allow an early therapeutic approach and a better prognosis.

Zhao et al. [119] highlighted how circulating EVs from ESCC patients are stable enough and are significantly higher in serum from ESCC patients compared with controls. In particular, when the cut-off value of EV number was set at 2.43 ng/mL, the sensitivity and the specificity corresponded to 75% and 85%, respectively. However, no significant association among EV number, gender, age, tumour size, lymph node invasion, metastasis, tumour grade, and UICC stage was detected. Takeshita and colleagues [120], isolated EVs from venous blood samples obtained by 101 ESCC patients and 46 healthy controls to perform miRNA profiling. Among the upregulated miRNAs, the expression level of miR-1246 in ESCC patients derived from EVs was significantly higher than in controls (*p* < 0.0001). Of note, the miR-1246 level significantly dropped after primary tumour resection, suggesting its specificity as a biomarker. The authors also demonstrated that the expression of miR-1246 correlates with tumour stage, lymph node status, metastatic burden, and with a 2-year OS. Patients expressing higher miR-1246 content have also a poor prognosis (Table 6).

Investigating plasma derived miRNAs on ESCC patients, a six-miRNA panel, including miR-106a, miR-18a, miR-20b, miR-486-5p, miR-584, and miR-223-3p, was identified by Zhou et al. [121] as differentially expressed in ESCC patients compared with controls. When combined, the miRNA signature displays a sensitivity varying from 85.3 to 92.5%, and a specificity ranging from 90.6 to 93.5%. Their expression in EVs demonstrated that miR-223-3p and miR-584 were dysregulated consistent with their plasma level. On the contrary, miR-20b and miR-486-5p were significantly downregulated in EVs from ESCC patients compared to healthy individuals. The authors demonstrated that, from among these miRNAs, only miR-486-5p was deregulated in the plasma, EVs, and tumour tissues. Interestingly, when the diagnostic potential of free plasma miRNAs and EV-miRNAs were compared, EV-miR-223-3p content displayed a higher diagnostic accuracy than plasma miR-223-3p (AUC corresponding to 854). Concerning the adenocarcinoma subtype, miRNA profiling allowed Warnecke-Ebertz et al. [122] to demonstrate the upregulation of miR-126-5p, miR-146a-5p, miR-192-5p, miR-196b-5p, miR-223-3p, miR-223-5p, miR-409-3p, and miR-483-5p in circulating EVs from EAC patients. Conversely, miR-22-3p, miR-23b-5p, miR-27b-3p, miR-149-5p, miR-203-5p, miR-224-5p, miR-452-5p, miR-671-3p, miR-944-5p, and miR-1201-5p were found to be downregulated (Table 6).

Stathmin-1 is a microtubule-destabilizing cytosolic phosphoprotein, which is post-transcriptionally regulated by miR-34a, miR-223, and miR-193b, and plays a central role in tumour cell proliferation and migration. Stathmin-1 is associated with metastatic disease and poor prognosis in osteosarcoma, prostate cancer, head and neck squamous cell carcinoma, hepatocellular carcinoma, colorectal cancer, gallbladder carcinoma, and non-small-cell lung cancer [123,124,125,126]. Likewise, in ESCC, stathmin-1 was linked to tumour invasiveness and is proposed as a predictor of poor prognosis. Stathmin-1 has been found enriched in EVs and proposed as a promising ESCC biomarker by Yan et al. [127]. By comparing nearly 1000 clinical samples, the authors demonstrated a discrimination power of 81% in sensitivity and 94% in specificity for ESCC. Raised concentrations of stathmin-1 were also associated with lymph node metastasis, even in the early stage. Since stathmin-1 is a cytoplasmic protein without a signal peptide, it is unclear how it is able to enter the bloodstream. Tumour-derived EVs were enriched in stathmin-1 compared with those from immortalized normal squamous oesophagus epithelial cell line (Het-1A), indicating that EV stathmin-1 content may be considered a surrogate of its intracellular levels, and therefore, a suitable tumour biomarker. The authors concluded that stathmin-1 is an outstanding diagnostic and predictive marker for squamous cell carcinoma, particularly for ESCC.

In a different study, a chimeric EV-mRNA, the seG-NchiRNA, was proposed as a biomarker to discriminate the early and advanced ESCC stage, as well as for the postoperative surveillance, therapeutic response, and tumour recurrence. The authors confirmed that the intracellular G-NchiRNA in ESCC cells closely correlated with the salivary EV-G-NchiRNA content. Moreover, it was reported that the G-NchiRNA increased with tumour growth, and that the amount of G-NchiRNA in tumour lysates significantly correlated with its content in salivary and serum EVs [128] (Table 6).

### 6.2. Gastric Cancer (GC)

Gastric cancer (GC) is a gastrointestinal malignancy with an overall poor prognosis, except for Early Gastric Cancer, a neoplasm involving the mucosa and the submucosa regardless of regional lymph nodes [164,165]. In fact, an 85% 5-year survival is reported for early gastric carcinoma. Similar to several cancers, onco-markers such as CEA and CA19.9 (also called GICA) have been employed in the clinical practice for diagnosis and, more importantly, for follow-up and prognostic purposes [166]. Yang et al. [167] identified the EV-miR-423-5p content as a novel marker for gastric cancer since it was found significantly higher in patients than in controls. Moreover, its level correlated with lymph node metastases and a high miR-423-5p level was associated with a poor prognosis. When compared to CEA or CA19.9, EV-miR-423-5p enrichment showed a more accurate diagnostic power. Moreover, Kumata et al. [139] have reported that EV-miR-23b content is significantly decreased in the plasma of GC patients. Depending on the tumour stage, it significantly correlates with the tumour size, a deep invasion, lymph node involvement, OS, and Disease-Free Survival (DFS). Therefore, the downregulation of miR-23b has been considered a potential marker for diagnostic purpose and for monitoring tumour relapse. EV-miR1246 content [120], already discussed for its potential diagnostic suitability in ESCC patients, was also found to be upregulated in GC patients’ EVs. Moreover, the properness of the EV-miR1246 level to discriminate the healthy controls from the early stage (stage I according to TNM staging system) GC patients was also reported. The EV-miRNA content has been also proposed for the GC staging. Zhang and colleagues [142] analysed EV-miRNA content in patients with GC at different stages: primary Gastric Cancer (pCG), GC with lymph node metastasis (GCln), GC with ovarian metastases (GCo), and GC with liver metastases (GCl). EV-miR-10b-5p, miR-101-3p, and miR-143-5p were recommended as potential biomarkers for GCln, GCo, and GCl, respectively. In patients with a primary tumour who underwent resection, a decreased expression of the miR-29 family members (miR-29a-3p, miR-29b-3p, and miR-29c-3p) in EVs recovered from peritoneal lavage fluid was indicated as a predictor of peritoneal recurrence, while miR-29s was proposed as a predictor of T4 cancer recurrence (tumours with serosa invasion) [143] (Table 6). Long non-coding RNAs (lncRNAs) are ncRNAs composed of 200 or more nucleotides contribute to the EV cargo. A diagnostic value of EV-lncUEGC1, HOTTIP, and lncRNA-GC1 content has been reported in GC. Zhao et al. [155] have proposed the increased EV-HOTTIP content as a potential GC biomarker by analysing its expression in the sera of 246 patients (126 GC samples and 120 controls). Moreover, EV-HOTTIP expression directly correlates with the tumour stage and invasion. The combination of EV-HOTTIP, CEA, CA19.9, and CA74-2 also ameliorated the diagnostic accuracy of CEA, CA19.9, and CA74-2. lncUEGC1 and lncUEGC2 were found to be upregulated in the plasma from early-stage GC patients. [154]. The level of lncUEGC1, but not lncUEGC2, was also found to be highly dependent on their EV content, being drastically reduced in EV-depleted plasma. The AUC was higher for lncUECG1 than lncUECG2 (0.8406 vs 0.6522). Again, the diagnostic potential of these EV biomarkers was higher than CEA or CA19.9. In a larger study by Guo et al. [157], involving 826 individuals, lncRNA-GC1 was found to discriminate GC in early GC patients, in healthy individuals with or without H. Pylori infection, and individuals with pre-malignant gastric lesions (intestinal metaplasia and atrophic gastritis) (Table 6). Although endoscopy is required for a conclusive diagnosis, it is conceivable to assume that the combination of standard biomarkers and EV-specific cargoes may be useful to better select patients requiring invasive diagnostic procedures.

### 6.3. Colorectal Cancer

Globally, more than 1,200,000 new colorectal cancer (CRC) cases are predicted yearly, which accounts for approximately 10% of all incident cancers. The mortality in CRC patients has been estimated to be approximately 609,000. In 2010, 141,570 new CRC cases and 51,370 deaths were estimated in U.S. Based on these data, CRC accounts for nearly 10% of cancer-related mortality in U.S. [100]. Moreover, more than 2.2 million new CRC cases and 1.1 million deaths are expected in 2030 [168]. CRC can be sporadic (the vast majority of incidents), familial, or hereditary. In the context of hereditary CRC, Familial Hereditary Polyposis (FAP) and Lynch syndrome are the most common genetic susceptibility syndromes influencing the development of CRC. In many countries, screening programs for CRC by Fecal Occult Blood Test (FOBT), procto-sigmoidoscopy, or colonoscopy are available. The FOBT was reported to display sensitivity and a specificity corresponding to 65–80% and 77.87–90.12%, respectively, based on the method used (guaiac or immunoassay test) [169]. Procto-sigmoidoscopy and colonoscopy have the ideal diagnostic accuracy, however, since they are invasive, they should be reserved for high-risk patients. Therefore, non-invasive and accurate diagnostic approaches including EVs have been proposed for high-risk patients. This was the aim of Ostenfeld et al. [135] who first described 13 dysregulated miRNAs in Ep-Cam positive EVs purified from peripheral blood samples of CRC patients and controls. Of note, among them, eight (miR-16-5p, miR-23a-3p, miR-23b-3p, miR-27a-3p, miR-27b-3p, miR-30b-5p, miR-30c-5p, and miR-222-3p) were reduced after surgery, suggesting their tumoural origin. Ogata-Kawata et al. [130] reported seven highly expressed miRNAs (miR-23a, miR-1246, let-7a, miR-1229, miR-150, miR-223, and miR-21) in CRC patients’ serum-derived EVs, which significantly decrease after surgical resection, again indicating their CRC origin. From among them, miRNAs, miR-23a, miR-1246, and miR-21 have shown the best diagnostic power (AUC corresponding to 0.953, 0.948, and 0.798, for miR-23a, miR-1246, and miR-21, respectively). Additionally, 11 miRNAs (miR-23a, miR-92a, miR-221, miR-301a, miR-31, miR-143, miR-142, miR-223, miR-18a, miR-135b, and miR-18b) were found to be dysregulated in serum EVs from CRC patients by Karimi et al. [136]. They selected miR-23a and miR-301a for further analyses and demonstrated that these miRNAs discriminate cancer patients from controls, based on the AUC values (0.900 and 0.840 for miR-23a and miR-301a, respectively). A lower diagnostic accuracy, but one worth mentioning, was reported for the enrichment of miR-486-5p [129] and miR-6803-5p [144] in EVs from CRC patients (Table 5).

The RNA sequencing of plasma EV cargo derived from CRC patients allowed Min et al. [131] to identify let-7b-3p, miR-139-3p, and miR-145-3p as potential CRC biomarkers (AUC = 0.927). miR-125a-3p was also described as being upregulated in the circulating EVs from CRC patients by Wang et. al. [145]. The AUC for miR-125a-3p corresponded to 0.685, which is generally considered unfulfilling. However, in combination with CEA, an increased diagnostic accuracy was reported (AUC = 0.855). The EV miR-150-5p content in combination with the serum CEA was also proposed as a diagnostic marker [141]. Among lncRNAs, lncUCA1, CCAT2, RPPH1, CRNDEh, GAS5, and HOTTIP have been reported as potential diagnostic biomarkers in CRC [156,158,159,160,161,170]. lncUCA1, GAS5, and HOTTIP have been found downregulated in CRC patients, while CCAT2, RPPH1, and CRNDEh were upregulated. CRNDEh showed a sensitivity of 70.3% and a specificity of 94.4%. Again, Liu et al. [170] demonstrated a diagnostic value superior to CEA. In a different study, Hu et al. [162] identified a panel of six EV-associated upregulated lncRNAs, (LNCV6_116109, LNCV6_98390, LNCV6_38772, LNCV_108266, LNCV6_84003, and LNCV6_98602) by screening ncRNAs in 50 CRC patients and 50 healthy individuals (Table 6). Several studies have revealed a differential expression of proteins in the circulating EVs from CRC patients. In a previous study, 36 proteins were found to be upregulated and 22 were downregulated in the circulating EVs from CRC patients [171]. Bavisotto et al. [151] also identified the heat shock protein 60 (Hsp60) in EVs recovered from CRC patients before and after the removal of primary tumours. Glypican-1 (GPC1)-positive circulating EVs from CRC patients have been proposed for diagnostic purposes [140]. The percentage of GPC1 (+) EVs was found to be markedly increased and normalized after surgery. CD147-positive EVs were also detected in the plasma of CRC patients with the AUC corresponding to 0.932 [150]. Moreover, the enrichment of Copine III (CPNE3) in the circulating EVs of CRC patients has been also proposed as diagnostic [35]. EV-CPNE3 content showed a better diagnostic power than CEA and, the combination of EV-CPNE3 and CEA was found to be superior to EV-CPNE3 or CEA to identify cancer patients (Table 6).

Lydic et al. [172] performed a lipidome profiling of EVs secreted by the colorectal cancer cell line LIM1215. However, no specific diagnostic marker was identified.

### 6.4. Pancreatic Cancer

Pancreatic cancer (PC) is the seventh leading cause of global cancer deaths in industrialized countries. PC is mainly divided into two subtypes: Pancreatic Adenocarcinoma and Pancreatic Neuro Endocrine Tumours (NETs). The pancreas adenocarcinoma is more aggressive than the NETs, with an OS corresponding to 24% 1 year after diagnosis, and 9% at 5 years. Different reasons can explain the poor prognosis. More specifically, the timing of the diagnosis, as 80–90% of patients present with an unresettable pancreatic tumour at diagnosis [6,173].

Given the highly aggressive behaviour and poor prognosis, the involvement of EVs in the pathogenesis, metastatic spread, diagnosis, prognosis, and PC treatment has been extensively investigated. Que et al. [132] first described the EV-miRNA content as a valuable diagnostic tool. They compared the EV-miRNA content by selecting 22 PC patients, 7 benign pancreatic tumour (BPT) patients, 6 patients suffering from chronic pancreatitis (CP), and 8 healthy participants (HP). Among the four initially selected miRNAs (miR-21, miR-17-5p, miR-155, and miR-196), miR21 and miR17-5p were further investigated to differentiate PC from non-PC. With the optimal cut-off value, sensitivities and specificities corresponded to 72.7% and 92.6% for miR-17-5p, and 95.5% and 81.5% for miR-21, respectively. They also compared early-stage versus advanced PC and found that miR17-5p may be a potential biomarker for advanced and unresettable PC. Subsequently, Machida et al. [137] have proposed saliva-derived EVs for biomarker discovery in pancreaticobiliary malignancies. When compared with controls, miR-1246 and miR-4644 obtained from saliva-derived EVs were found upregulated. Statistical analyses revealed a sensitivity of 75% and a specificity 76% for miR-4644. For miR-1246, the results yielded an AUC corresponding to 0.814, a sensitivity of 66.7%, and a specificity of 100%. Their combination further improved the sensitivity up to 83.3%. The analysis of circulating EV-miRNA content in 40 patients (29 with PC and 11 healthy controls or chronic pancreatitis) allowed Lai et. al. [133] to propose the upregulation of miR-10b, miR-21, miR-30c, and miR-18 and the downregulation of miR-let7a and miR-122 as diagnostics for PC. miR-1246, along with miR-196s, was also described by Xu et al. [138] as potential markers for localized PC, since their upregulation selectively discriminate early stage (stage I and IIA) PC patients from controls (AUC of 0.73 for miR-1246 and of 0.71–0.81 for miR-196). These observations sustain that EV-miRNA cargo could distinguish PC from other pancreatic lesions. In a different study, EV-miR-191, miR-21, and miR-451a were found significantly upregulated in patients with PC and Intraductal Papillary Mucinous Neoplasia (IPMN) compared to the control group [146], thus suggesting they could be considered excellent prognostic markers, not only for pancreatic adenocarcinoma, but also for cystic pancreatic neoplasms. In the same study, miR-451 was correlated to IPMN mural nodules and considered feasible to assess the risk of progression to invasive cancer. Moreover, miR-21 was proposed as an independent PC prognostic factor (Table 6). Different RNAs were evaluated by Kitagawa et al. [149]. Four mRNAs (mRNAs: CCDC88A, ARF6, Vav3, and WASF2) and five small nucleolar RNAs (snoRNAs) (SNORA14B, SNORA18, SNORA25, SNORA74A, and SNORD22) were analysed in circulating EVs from patients with PC and controls. The AUC for WASF2, ARF6 mRNAs, SNORA74A, and SNORA25snoRNAs were >0.9 in distinguishing PC patients from controls, therefore displaying a diagnostic accuracy greater than Ca19.9 (Table 6). As far as EV protein-based diagnostic strategies were considered, EV proteins such as carcinoembryonic antigen-related cell adhesion molecules (CEACAMs), Tenascin C, Glypcan-1, and ZIP-4 have been proposed as biomarkers. CECAMS 1/5 and Tenascin C were evaluated in EVs recovered from the pancreatic ductal fluid of PC patients. The authors found that more than 2000 EV proteins were able to discriminate between malignant and benign diseases [152]. Jin et al. [153] analysed the ZIP-4 (a membrane protein) EV level in 24 PC patients and 32 patients with non-neoplastic pancreas disease or healthy controls. PC-derived EVs were found to be enriched in ZIP-4 with the AUC corresponding to 0.8931 (PC vs non-PC). The EV-Glypcan-1 (GCP-1) content was found to be enriched (*p* < 0,0001) in EVs recovered from a large study cohort, including 190 PC patients and 100 controls [134]. Moreover, GCP-1 was also described as a potential marker of early pancreatic ductal adenocarcinoma with a diagnostic accuracy superior to CA19.9. The authors suggested EV-Glypcan-1 as appropriate for all PC stages (Table 6). Finally, more recently, Tao et al. [174] demonstrated the presence of 20 dysregulated phospholipids in PC compared to controls, by using lipidomic analysis. Among those, three lipids were found significantly associated with patient clinical-pathologic features. LysoPC 22:0 was associated with tumour stage, while CA19-9 and CA242 were associated with tumour diameter and lymphocyte count. In addition, Plasmenyl-PC 36:0 was associated with tumour stage, CA19-9, CA242, CEA, and lymphocyte count, while PE 34:1; PE (16:0/18:1) was found to correlate with the patient’s OS.

### 6.5. Hepatocellular Carcinoma

Hepatocellular carcinoma (HCC) is a primary liver cancer that usually develops in the context of chronic liver diseases [175]. In addition, chronic hepatitis, HBV or HCV infection, alcohol consumption, and liver cirrhosis are considered to be the most relevant associated risk factors. Primary liver cancer is the fourth leading cause of cancer-related mortality worldwide, with over 780,000 deaths in 2018. Liver cancer is the second most lethal tumour after PC, exhibiting an 18% five-year survival. Diagnosis is usually made upon clinical suspicion or surveillance in high-risk patients by serum alpha-fetoprotein (AFP) and imaging (liver ultrasound generally followed by contrast-enhanced CT scan and/or MRI) [176]. However, AFP is not specific for HCC, since an increased level of AFP can be also found in benign liver diseases. This implies that the discovery of new non-invasive biomarkers may be useful for the early diagnosis and management of HCC. The application of the EV-based diagnosis in HCC was first explored by Wang et al. [177]. The circulating EV level was evaluated in patients with HCC (*n* = 55), liver cirrhosis (*n* = 40), and healthy subjects (*n* = 21). EVs were found statistically higher in HCC patients than in patients with liver cirrhosis and healthy participants (*p* < 0.001). Circulating EV level correlated with tumour size and stage (*p* < 0.01), according to the HCC TNM staging. In HCC patients, the EV number was found to be reduced one month after surgical resection, suggesting that they are directly produced by cancer cells. Moreover, ROC analysis demonstrated a better performance of EV level than AFP for early HCC detection. The AUC was able to discriminate patients with early HCC from liver cirrhosis (0.83) to stage I HCC and (0.94) to stage II HCC. More recently, Julich-Heartel et al. [36] investigated the diagnostic potential of EV profiling in liver cancers. They purified AnnexinV^+,^EpCAM^+^ CD147^+^ EVs from patients with HCC and Cholangiocarcinoma (CCA) (*n* = 127), with liver cirrhosis (*n* = 54), and negative controls (*n* = 202). The AnnexinV^+^/EpCAM^+^/CD147^+^ EVs were correlated with the tumour burden, and were able to differentiate HCC and CCA from healthy individuals and patients with cirrhosis. Moreover, the authors proposed a diagnostic algorithm for patients at high risk for liver cancer also including EV characterization. Wang et al. [178] exploited a Tethered Lipoplex Nanoparticle (TLN) assay to capture and analyse the EV cargo and related antigens. They compared AFP and Glypcan-3 (GPC-3) EV-mRNA content to plasma AFP level in 40 HCC patients and 38 controls. The combinations of AFP- and GPC-3-miRNA showed the highest diagnostic accuracy with a positive predictive value (PPV) and a negative predictive value (NPP) corresponding to 100% and 95% respectively. Moreover, EV-AFP-mRNA was found to be more stable than plasma AFP, thus suggesting their potential application in clinical practice (Table 6). In patients with HBV infection, who should be monitored every 6 months for the risk of HCC, EV-miR-212 content was found to be highly relevant [147]. For the HBV-infection related HCC, EV-miR-212 content showed a higher AUC than AFP (0.886 vs. 0.849). EV-miR-21, one of the onco-miRNAs frequently upregulated in solid cancers, was found to be upregulated in HCC patients compared with tumour-free Chronic Hepatitis B (CHB patients) with a *p* < 0.0001 [148]. The EV-miR-21 level also correlated with tumour stage and cirrhosis but not with other clinical-pathologic features. Of note, the diagnostic value of the EV-miR-21 content was higher than the free circulating miR-21. In a different study, miR-15b-5p, miR-338-5p, and miR-764 were found to be upregulated in HCC patients and were able to discriminate HCC from cirrhosis and healthy subjects [163] (Table 6). Besides mRNAs, several lncRNAs have been implicated in the development and progression of liver cancers, while only some of them likely display diagnostic relevance. To assess the feasibility of using EV-lncRNA cargo as a diagnostic tool, Yao et al. [179] investigated circulating EVs enriched in lnc-GPR89B-15, lnc-FAM72D-3, lncEPC1-4, and lnc-ZEB2-19. Promising results were obtained particularly for lnc-ZEB2-19 which exhibited a higher AUC value in detecting HCC compared to healthy subjects (AUC = 0.852) (Table 6).

Chapuy-Regaud et al. [180] performed a lipidomic analysis on EVs released from uninfected and HEV-infected cells and showed a differential amount of free cholesterol, ceramides, phosphatidylserine, sphingomyelin, and phosphoinositides. Similarly, Haraszti et al. [77] analysed the lipid composition in EVs from Huh7 hepatocellular carcinoma cells and human MSCs and did not find significant differences.

Overall, these results strengthen the notion that EV-related markers may strongly influence the diagnosis of liver disease and early liver cancer compared to conventional biomarkers such as AFP.

## 7. Conclusions

Modern medicine and research strives to move towards personalised diagnostics and therapeutics to offer the best therapeutic options. The so-called “bench to the bedside” research holds great promise in advancing science and clinical medicine. This is particularly true for basic and clinical oncology where EVs have gained particular interest. Concerning cancer diagnosis, there is a growing interest towards non-invasive techniques. Non-invasive diagnostic tools would be better tolerated by patients and to old, frail, and medically complex patients. In oncology, liquid biopsy represents the most feasible non-invasive procedure for tumour diagnosis and genotyping aimed to identify tailored therapeutic approaches and to monitor the response to therapy. Circulating Cancer Cells (CCCs) have been largely studied for diagnostic purposes using liquid biopsy. CCCs may not only support cancer diagnosis but also represent a potential prognostic tool, since in many cancers, the burden of CCCs seems to correlate with the survival and the rate of relapse. However, CCCs are little-represented in the bloodstream and their proper isolation is complex. On the contrary, EVs are abundant in peripheral blood as well in many other biological fluids and it is becoming even more clear that cancer-derived EVs can be easily isolated and discriminated from non-cancer-derived EVs. Moreover, these peculiar features, and their stability in biological samples could overcome several obstacles faced by clinicians when a liquid biopsy is exploited. EVs-based diagnosis holds several advantages even when compared to free tumour related products such as DNA, mRNAs, miRNAs, or proteins (Table 3, Table 4, Table 5 and Table 6). The encapsulation of cancer-derived molecules into EVs exerts protection against circulating degrading enzymes and translates into a more reliable and replicable diagnostic approach. Moreover, the diagnostic power of EVs particularly increases when combined with existing biomarkers, as reported for AFP for HCC, CEA, and CA19.9 for gastrointestinal cancers, to name a few. Overall, as reported by the number of ongoing clinical trials (https://www.clinicaltrials.gov/ct2/results?recrs=&cond=Cancer&term=exosomes&cntry=&state=&city=&dist= (accessed on 10 April 2021), EVs should be considered good candidates to revolutionize cancer diagnosis, particularly at the early stage. However, larger studies are warranted to allow their transfer in the clinical practice.

## Figures and Tables

**Table 1:** EV isolation methods.

Method	Brief Technique Description	Advantages	Disadvantages	Ref.
UC	The sample is centrifuged (300× *g* for 10 min; 2000× *g* for 20 min to eliminate dead cells; and 10,000× *g* for 30 min to remove debris). EVs are isolated by UC at 100,000× *g* for 60 min at 4 °C. The pellet is resuspended in PBS and re-centrifuged at 100,000× *g* for 60 min	It represents the most widely used technique for EV isolation in basic and translational research. For these reasons it is highly standardized and reproducible	Generation of EV aggregates, contamination of the EV sample with smaller size particles. The whole isolation process is time consuming	[16] [17] [18] [19]
DG	A density gradient is generated by adding decreasing concentration of iodixanol or sucrose in a tube. The sample is then added on the top of the gradient and is centrifuged at 100,000× *g* for 18 h at 4 °C. After centrifugation, the recovered gradient fractions are diluted in PBS. An additional 3 h centrifugation is performed at 100,000× *g*	It represents a highly efficient method allowing to isolate purified EVs	It is more time consuming and more labour- intensive than standard UC. This consideration can make its introduction in the clinical practice difficult	[16] [17]
SEC	SEC separates molecules based on their size by filtration through a resin-packed column. After sample centrifugation (1100× *g* for 10 min) and filtration, samples are loaded in size exclusion chromatography columns	SEC isolates high purity, functionally and structurally preserved EVs. It has been defined as one of the best methods for EV isolation	Compared to other methods, the total EV yield is lower and in the clinical setting, higher volumes of biological fluid may be needed to overcome this issue	[18,20]
Polymer-based precipitation (e.g.,: Exoquick)	The method is based on the use of PEG to capture EVs. After PEG preparation, the sample undergoes isolation using 10% PEG solution at 4 °C for 2 h. After incubation, samples are centrifuged at low-speed × 10 min. To ensure major purity a double-step approach can be followed	It represents a fast, easy, and widespread method for EV isolation. It also easily allows a simultaneous isolation from multiple samples	EV obtained with this method have higher chances of being contaminated and therefore less suitable for omics-based analysis	[14,20,21]
Immunoaffinity capture	The immunoaffinity capture method relies on the isolation of EVs based on the expression of surface markers. It commonly uses antibodies against specific EV surface proteins, e.g., tetraspanin: CD9, CD63, and CD81	The immunoaffinity capture isolation allows EV-specific isolation based on their surface markers. It can also be used as an additional step after UC to enhance EV purity	The removal of antibodies from EV surface could damage EVs. The selection of specific EVs could eventually not reflect the characteristics of all separated EVs. It is more expensive compared to other methods.	[17,20]
Microfluidic isolation	It consists in a high-throughput method using microfluidic devices to isolate EVs based on several principles: immunoaffinity, size, and density. The most used is the immuno-microfluidic technique, which is similar to the immune-affinity-capture isolation method. Antibodies immobilized on microfluidic devices serve to EV isolation	Relatively fast and high purity EV isolation can be obtained with this technique. It is a new and promising method for EV isolation	Shares with immunoaffinity the same disadvantages. It is expensive and standardization is still lacking	[18]

UC: Ultracentrifugation, DG: Density Gradient Centrifugation, SEC: Size Exclusion Chromatography.

**Table 2:** EV characterization methods.

Method	EV Information	Disadvantages	Ref.
TEM	Structure Shape Size Using gold particle conjugate Ab can allow the identification of EV specific markers	Does not allow precise quantification Requires trained personnel Not widely available	[21,22,23]
AFM	Structure (is able to provide sub-structural information) Shape Size	Does not allow precise quantification Requires trained personnel Not widely available	[22,23]
DLS	Size distribution Quantification	Can misrepresent EV concentration in non-processed or complex biofluids	[24]
NTA	Size distribution Quantification	Can misrepresent EV concentration in non-processed or complex biofluids	[24]
WB	EV specific proteins	Does not allow quantification	[27]
PCR	Based on the used method (e.g.,: PCR vs RT-PCR) can identify specific nucleic acids in the EV cargo	Standard PCR could be inappropriate if starting nucleic acid concentration is too low	[25,28,29]
FC	Specific markers on EVs New generation FC is able to quantify EVs	Old generation FC can only analyze bead conjugated EVs	[25,26]
OMICS based techniques	Allow EV DNA, RNA, protein, and lipid profiling	High costs and resource availability	[25,30,31]

TEM: Transmission Electron Microscopy, AFM: Atomic Force Microscopy, DLS: Dynamic Light Scattering, NTA: Nanoparticle Tracking Analysis, WB: Western Blot, ELISA: Enzyme linked immune-adsorbent protein assay, PCR: Polymerase Chain Reaction, FC: Flow Cytometry.

**Table 3:** EVs as potential biomarkers in CNS cancer.

Biomarker Types	Specific Markers	Tumour	Expression	Ref.
**miRNAs**	miR-301a	GBM	Upregulated	[47]
	miR-182-5p	GBM	Upregulated	[48]
	miR-328-3p	GBM	Downregulated	[48]
	miR-339-5p	GBM	Downregulated	[49]
	miR-340-5p	GBM	Downregulated	[48]
	miR-485-3p	GBM	Downregulated	[48]
	miR-486-5p	GBM	Upregulated	[48]
	miR-543	GBM	Downregulated	[48]
	miR-320	GBM	Upregulated	[49]
	miR-301a	GBM	Upregulated	[47]
	miR-182-5p	GBM	Upregulated	[48]
	miR-328-3p	GBM	Downregulated	[48]
	miR-339-5p	GBM	Downregulated	[48]
	miR-340-5p	GBM	Downregulated	[48]
	miR-485-3p	GBM	Downregulated	[48]
	miR-486-5p	GBM	Upregulated	[48]
	miR-543	GBM	Downregulated	[48]
	miR-320	GBM	Upregulated	[49]
	miR-574-3p	GBM	Upregulated	[49]
	miR-21 miR-21	GBM GBM/Brain metastases	Upregulated Upregulated	[50] [51]
	miR-218	GBM	Upregulated	[50]
	miR-193b	GBM	Upregulated	[50]
	miR-331	GBM	Upregulated	[50]
	miR-374a	GBM	Upregulated	[50]
	miR-548c	GBM	Downregulated	[50]
	miR-520f	GBM	Downregulated	[50]
	miR-27	GBM	Downregulate	[50]
	miR-130b	GBM	Downregulated	[50]
	miR-222	GBM/Brain metastases	Upregulated	[51]
	miR-124-3p	GBM/Brain metastases	Upregulated	[51]
**mRNAs**	EGFRvIII	GBM	Upregulated	[52,53]
	Syndecan-1 (SDC1)	GBM	Upregulated	[54]
	p65	GBM	Upregulated	[55]
	DNM3	GBM	Upregulated	[55]
	CD117	GBM	Upregulated	[55]
	PTEN	GBM	Downregulated	[55]
	p53	GBM	Downregulated	[55]
	APC	GBM	Downregulated	[55]
	RNU6-1	GBM	Upregulated	[49]
	IDH1	GBM	Upregulated	[56]
	KIAA1549/BRAF	Pilocytic astrocytoma	Upregulated	[57]
	c-MET	Medulloblastoma	Upregulated	[58]
	ABCB1	Medulloblastoma	Upregulated	[58]
	MMP2	Medulloblastoma	Upregulated	[58]
	BSG	Medulloblastoma	Upregulated	[58]
	ITG-A9	Medulloblastoma	Upregulated	[58]
**Proteins**	EGFR EGFRvIII	GBM GBM	Overexpressed Overexpressed	[59] [59]
	PDPN	GBM	Overexpressed	[59]
	IDH1 R132H	GBM	Overexpressed	[59]
	PTRF	GBM	Ratio	[60]
	vWF	GBM	Overexpressed	[61]
	AZGP1	GBM	Overexpressed	[61]
	Serpin3	GBM	Overexpressed	[61]
	FTL	GBM	Overexpressed	[61]
	C3	GBM	Overexpressed	[61]
	APOE	GBM	Overexpressed	[61]
	CD63	GBM	Ratio	[62]
	FOLR1	NFPA	Downregulated	[63]
	EpCAM	NFPA	Downregulated	[63]

GMB: Glioblastoma, NFPA Non Functional Pituitary Adenoma.

**Table 4:** EVs as potential biomarkers in head and neck cancer.

Biomarker Types	Specific Markers	Tumour	Expression	Ref.
**miRNAs**	miR-21 miR-21	LSCC OSCC	Upregulated Upregulated	[95] [94]
	miR-27b	OSCC	Upregulated	[92]
	miR-27a-3p	OSCC	Upregulated	[86]
	miR-412-3p	OSCC	Upregulated	[86]
	miR-512-3p	OSCC	Upregulated	[86]
	miR-302b-3p	OSCC	Upregulated	[86]
	miR-517b-3p	OSCC	Upregulated	[86]
	miR-494-3p	OSCC	Upregulated	[86]
	miR-24-3p miR-24-3p	OSCC NPC	Upregulated Upregulated	[88] [96]
	miR-31	OSCC	Upregulated	[93]
	miR-184	OSCC	Upregulated	[94]
	miR-145	OSCC	Downregulated	[94]
	Let-7b-5p	NPC	Upregulated	[96]
	miR-140-3p	NPC	Upregulated	[96]
	miR-192-5p	NPC	Upregulated	[96]
	miR-223-3p	NPC	Upregulated	[96]
**LncRNA**	HOTAIR	LSCC	Upregulated	[95]
**Proteins**	CD9	OC	Downregulated	[84]
	CD81	OC	Downregulated	[84]
	CYPA	EBV-associated NPC	Overexpressed	[97]

LSCC: Laryngeal Squamous Cell Carcinoma, OSCC: Oral Squamous Cell Carcinoma, NPC Nasopharyngeal Carcinoma.

**Table 5:** EVs as potential biomarkers in lung cancer.

Biomarker Types	Specific Markers	Tumour	Expression	Ref.
**miRNAs**	miR-182	LC	Upregulated	[113]
	miR-21-5p	LAC	Upregulated	[104]
	miR-520c-3p	LC	Upregulated	[107]
	miR-19b-3p	LAC	Upregulated	[104]
	miR-221-3p	LAC	Upregulated	[104]
	miR-502-5p	LAC	Upregulated	[105]
	miR-376a-5p	LAC	Upregulated	[105]
	miR-1974	LAC	Upregulated	[105]
	miR-378a	LAC	Upregulated	[105]
	miR-379	LAC	Upregulated	[105]
	miR-151a-5p	LAC	Upregulated	[105]
	miR-139-5p	LAC	Upregulated	[105]
	miR-200b-5p	LAC	Upregulated	[105]
	miR-190b	LAC	Upregulated	[105]
	miR-30a-3p	LAC	Upregulated	[105]
	miR-629	LAC	Upregulated	[105]
	miR-17	LAC	Upregulated	[105]
	miR-100	LAC	Upregulated	[105]
	miR-154-3p	LAC	Upregulated	[105]
	miR-505-5p	LAC	Upregualated	[106]
	miR-382-3p	LAC	Downregulated	[106]
	miR-1274b	LC	Upregulated	[107]
	miR-1-3p	LC	Downregulated	[111]
	miR-144-5p	LC	Downregulated	[111]
	miR-150-5p	LC	Upregulated	[112]
	miR-210	LC	Upregulated	[113]
**Proteins**	EGFRvIII	LC	Downregulated	[36]
	CD9	LC	Overexpressed	[36]
	TAG72	LC	Overexpressed	[36]
	MUC	LC	Overexpressed	[36]
	CD142	LC	Overexpressed	[36]
	N-cadherin	LC	Overexpressed	[36]
	CD163	LC	Overexpressed	[36]
	CD63	LC	Overexpressed	[36]
	CD81	LC	Downregulated	[36]
	TSG101	LC	Downregulated	[36]
	Hsp90	LC	Downregulated	[36]
	EpCam	LC	Downregulated	[36]

LC: Lung Cancer; LAC: Lung Adenocarcinoma.

**Table 6:** EVs as potential biomarkers in gastrointestinal cancer.

Biomarker Types	Specific Markers	Tumour	Expression	Ref.
**miRNAs**	miR-486-5p	CRC	Upregulated	[129]
	miR-21	CRC PC HCC	Upregulated Upregulated Upregulated	[130] [131,132,133] [134]
	miR-27a-3p	CRC	Upregulated	[135]
	miR-27b-3p	CRC EAC	Upregulated Downregulated	[135] [122]
	miR-222-3p	CRC	Upregulated	[135]
	miR-31	CRC	Upregulated	[136]
	miR-145-3p	CRC	Upregulated	[131]
	Let-7a	PC CRC	Downregulated Upregulated	[133] [130]
	miR-192-5p	EAC	Upregulated	[122]
	miR-223	CRC EAC	Upregulated Upregulated	[130] [122]
	miR-223-3p	ESCC EAC	Downregulated Upregulated	[121] [122]
	miR-223-5p	EAC	Upregulated	[122]
	miR-1246	ESCC CRC PC	Upregulated Upregulated Upregulated	[120] [130] [137,138]
	miR-106a	ESCC	Upregulated	[121]
	miR-18	PC	Upregulated	[133]
	miR-18a	ESCC	Upregulated	[121]
	miR-20b	ESCC	Downregulated	[121]
	miR-584	ESCC	Upregulated	[121]
	miR-126-5p	EAC	Upregulated	[122]
	miR-146a-5p	EAC	Upregulated	[122]
	miR-196	PC	Upregulated	[138]
	miR-196b-5p	EAC	Upregulated	[122]
	miR-409-3p	EAC	Upregulated	[122]
	miR-483-5p	EAC	Upregulated	[122]
	miR-22-3p	EAC	Downregulated	[122]
	miR-23a	CRC	Upregulated	[130]
	miR-23a-3p	CRC	Upregulated	[135]
	miR-23b	GC	Downregulated	[139]
	miR-23b-3p	CRC	Upregulated	[135]
	miR-23b-5p	EAC	Downregulated	[122]
	miR-149-5p	EAC	Downregulated	[122]
	miR-203-5p	EAC	Downregulated	[122]
	miR-224-5p	EAC	Downregulated	[122]
	miR-452-5p	EAC	Downregulated	[122]
	miR-671-3p	EAC	Downregulated	[122]
	miR-944-5p	EAC	Downregulated	[122]
	miR-1201-5p	EAC	Downregulated	[122]
	miR-423-5p	GC	Upregulated	[122]
	miR-139-3p	CRC	Upregulated	[140]
	miR-30b-5p	CRC	Upregulated	[135]
	miR-30c	PC	Upregulated	[133]
	miR-30c-5p	CRC	Upregulated	[135]
	miR-17-5p	PC	Upregulated	[132]
	miR-150	CRC	Upregulated	[130]
	miR-150-5p	CRC	Downregulated	[141]
	miR-10b	PC	Upregulated	[133]
	miR-10b-5p	GC	Upregulated	[142]
	miR-101-3p	GC	Upregulated	[142]
	miR-143-5p	GC	Upregulated	[142]
	miR-29	GC	Downregulated	[143]
	miR-29a-3p	GC	Downregulated	[143]
	miR-29b-3p	GC	Downregulated	[143]
	miR-29c-3p	GC	Downregulated	[143]
	miR-16-5p	CRC	Upregulated	[135]
	miR-1229	CRC	Upregulated	[130]
	miR-6803-5p	CRC	Upregulated	[144]
	miR-125a-3p	CRC	Upregulated	[145]
	miR-155	PC	Downregulated	[132]
	miR-4644	PC	Upregulated	[137]
	miR-122	PC	Downregulated	[133]
	miR-191	PC/IPMN	Upregulated	[146]
	miR-451a	PC/IPMN	Upregulated	[146]
	miR-212	HBV-related HCC	Upregulated	[147]
	miR-15b-5p	HCC	Upregulated	[148]
	miR-338-5p	HCC	Upregulated	[148]
	miR-764	HCC	Upregulated	[148]
**mRNAs**	ARF6	PC	Upregulated	[149]
	Vav3	PC	Upregulated	[149]
	WASF2	PC	Upregulated	[149]
**Proteins**	EpCAM	HCC/Colangiocarcinoma	Overexpressed	[36]
	Stathmin 1	ESCC	Overexpressed	[127]
	CD147	CRC HCC/Colangiocarcinoma	Overexpressed Overexpressed	[150] [36]
	Hsp60	CRC	Overexpressed	[151]
	Glypican-1 (GPC1)	CRC	Overexpressed	[140]
	CopineIII (CPNE3)	CRC	Overexpressed	[35]
	CEACAMs	PC	Overexpressed	[152]
	Tenascin C	PC	Overexpressed	[152]
	Glypcan-1 (GCP-1)	PC	Overexpressed	[134]
	ZIP-4	PC	Overexpressed	[153]
	AnnexinV	HCC/Colangiocarcinoma	Overexpressed	[36]
**lncRNAs**	lncUEGC1	GC	Upregulated	[154]
	HOTTIP	GC CRC	Upregulated Downregulated	[155] [156]
	lncRNAGC1	GC	Upregulated	[157]
	UCA1	CRC	Downregulated	[158]
	GAS5	CRC	Downregulated	[159]
	CCAT2	CRC	Upregulated	[160]
	RPPH1	CRC	Upregulated	[161]
	CRNDEh	CRC	Upregulated	[132]
	LNCV6_116109	CRC	Upregulated	[162]
	LNCV6_98390	CRC	Upregulated	[162]
	LNCV6_38772	CRC	Upregulated	[162]
	LNCV_108266	CRC	Upregulated	[162]
	LNCV6_84003	CRC	Upregulated	[162]
	LNCV6_98602	CRC	Upregulated	[162]
	GPR89B	HCC	Upregulated	[163]
	FAM72D-3	HCC	Upregulated	[163]
	EPC1-4	HCC	Downregulated	[163]
	ZEB2-19	HCC	Downregulated	[163]

CRC: Colorectal Cancer, PC: Pancreatic Cancer, IPMN: Intraductal Mucinous Papillary Neoplasm, HCC: Hepatocellular Carcinoma, EAC: Esophageal Adenocarcinoma, ESCC: Esophageal Squamous Cell Carcinoma, GC: Gastric Cancer.

## Data Availability

No new data were created or analyzed in this study. Data sharing is not applicable to this article.
